# An orchestrating role of mitochondria in the origin and development of post-traumatic stress disorder

**DOI:** 10.3389/fphys.2022.1094076

**Published:** 2023-01-10

**Authors:** Oleh Lushchak, Olha Strilbytska, Alexander Koliada, Kenneth B. Storey

**Affiliations:** ^1^ Department of Biochemistry and Biotechnology, Vasyl Stefanyk Precarpathian National University, Ivano-Frankivsk, Ukraine; ^2^ Research and Development University, Ivano-Frankivsk, Ukraine; ^3^ Institute of Food Biotechnology and Genomics, NAS of Ukraine, Kyiv, Ukraine; ^4^ Department of Biology, Carleton University, Ottawa, ON, Canada

**Keywords:** post-traumatic stress disorder, reactive oxygen species, mitochondria, inflammation, glucocorticoids

## Abstract

Post-traumatic stress disorder (PTSD) is one of the most discussed and actively researched areas in medicine, psychiatry, neurophysiology, biochemistry and rehabilitation over the last decades. Multiple causes can trigger post-traumatic stress disorder. Humans subjected to violence, participants in hostilities, victims of terrorist attacks, physical or psychological persecution, witnessing scenes of cruelty, survival of natural disasters, and more, can strongly affect both children and adults. Pathological features of post-traumatic stress disorder that are manifested at molecular, cellular and whole-organism levels must be clearly understood for successful diagnosis, management, and minimizing of long-term outcomes associated with post-traumatic stress disorder. This article summarizes existing data on different post-traumatic stress disorder causes and symptoms, as well as effects on homeostasis, genetic instability, behavior, neurohumoral balance, and personal psychic stability. In particular, we highlight a key role of mitochondria and oxidative stress development in the severity and treatment of post-traumatic stress disorder. Excessive or prolonged exposure to traumatic factors can cause irreversible mitochondrial damage, leading to cell death. This review underlines the exceptional importance of data integration about the mechanisms and functions of the mitochondrial stress response to develop a three-dimensional picture of post-traumatic stress disorder pathophysiology and develop a comprehensive, universal, multifaceted, and effective strategy of managing or treatment post-traumatic stress disorder.

## 1 Introduction

Dysregulation of organismal reactions to stress in humans and other mammals can lead to serious health problems. In recent times, traumatic events worldwide have included new highly pathogenic viruses, prions, terrorist acts, endless armed conflicts, and drastic natural disasters, occurring all over the planet and having a significant impact on lifestyle, behavior, and mental health (Arnberg et al., 2013). All these events and factors are highly traumatic, causing millions of people to undergo physical and/or mental injuries, risking their health and lives and becoming witnesses to violence and cruelty. Many different types of trauma can trigger post-traumatic stress disorder (PTSD) (Dutheil et al., 2021). Nowadays the term PTSD is understood as a multi-etiological, systemic, chronic disease, most often connected with the malfunction of blood vessels, metabolic disorders, and the immune system, among other factors (Sareen, 2014).

The diagnosis of PTSD is a long-lasting malfunction of an organism, originating from shocking, scary, or dangerous events or experiences (Wimalawansa, 2014) and can be explained as an interaction of a person with a traumatic factor (TF). Such events lead to inadequate reactions to environmental stressors, collectively known as « anxiety disorder». This complex syndrome is categorized as “Trauma- and Stressor-Related Disorders” in the Diagnostic and Statistical Manual of Mental Disorders version V. Traumatic factors build the base for PTSD initiation. Mental processing of the TF or event by the neuro-humoral interactions, and processes of gene expression cause the devastating functional impairment that accompanies PTSD. The diversity of TFs experienced can lead to considerable predictability of PTSD susceptibility and symptom severity. It is noteworthy that four (or more) interacting TFs greatly increase the danger of acquiring PTSD, as compared with three or less traumatic experiences (Benjet et al., 2016).

Surveys indicate that approximately 8% of all people experience PTSD although the experience of distress after a traumatic event does not necessarily warrant a diagnosis of PTSD. The probability of PTSD after a traumatic event can fluctuate from 1% to more than 50% (Spoont et al., 2015). Across the world, the lifetime prevalence of PTSD varies from 1.3% to 12.2% with 1-year prevalence rates varying from .2% to 3.8% (Karam et al., 2014). On the other hand, the International Classification of Diseases (ICD) interprets PTSD as one of the psychiatric disorders, connected with plenty of others by the same symptoms.

Existing evidence demonstrates significant physiological and metabolic effects of PTSD on humans at both cellular and organismal levels. Patients with PTSD are often characterized by elevated glucose, insulin, and creatinine (Michopoulos et al., 2016). The biomarkers of PTSD include the activity levels of certain genes, amounts of key proteins in the blood, levels of metabolites involved in energy processing, and levels of circulating microRNAs (Michopoulos et al., 2016). Fatty acids involved in neuroprotection were found to be reduced in the plasma of PTSD patients (Konjevod et al., 2019). These fatty acids including linolenic, linoleate, docosahexaenoic, eicosapentaenoic, and docosapentaenoic acids block the action of the NF-kB transcription factor and, in turn, reduce the generation of reactive oxygen species (ROS) (Mocking et al., 2018). Mitochondria are an important source of ROS in mammalian cells. However, ROS may damage mitochondria as part of the pathophysiology of numerous diseases (Murphy, 2009). The present review is focused on the basic mechanisms of mitochondrial functioning under PTSD conditions. We specifically focus on the contribution of mitochondrial dysfunction to systemic physiological (dys) regulation. Efforts to elucidate the causes of PTSD and formulate the role of mitochondria pertaining to PTSD-mediated symptomatology form the empirical foundation for developing an alternative therapy to target mitochondrial dysfunction for the prevention of risk associated with PTSD.

## 2 Etiology and symptoms of PTSD

Without adequate quantity and quality of treatment, PTSD can transform into a chronic disease. This syndrome is multi-faceted and includes a cellular reorganization of oxidative, ion, and protein metabolism that results in a malfunction of mental processes. PTSD can be caused by long-lasting distress, fear, shock, horror, or helplessness (van der Meer et al., 2017). Terrifying events result in flashbacks, nightmares and anxiety, as well as uncontrollable thoughts about the event (Mann and Marwaha, 2022). Many factors and situations can catalyze this process: e.g., armed conflicts (inside a country or between countries); industrial, technical, or social accidents, resulting in serious effects; terrorist acts, attacks, and threats; any kind of abuse; personal assault or being a witness of crime; difficult or incurable medical diagnosis, etc.

The origin and characteristics of PTSD development depends on an individual’s history of mental disease, the nature and type of trauma, and the experiences of each person. For many patients, PTSD, once formed, becomes chronic, and can last for many years. In a group of such patients, a joint course and development of both physical (somatic) and mental disorders are often observed. These include premature and/or early manifestation of aging symptoms, such as chronic pain syndrome, metabolic syndrome, neurocognitive impairment, states of obsessive fears and panic attacks, and dementia. Predisposition to PTSD can originate from neuroendocrine, inflammatory, metabolic, and transcriptional perturbations. At present, it is known that there is a significant correlation of PTSD with various cellular and molecular events including expression of nuclear or mitochondria DNA; epigenetic regulation of gene expression or silencing; neuroendocrine factors; and markers of inflammation (Passos et al., 2015). Enhancements of different DNA damage types, dysfunction of repair systems, telomere shortening, and modification of N-glycosylation profiles are the main factors that accelerate the aging process under PTSD. Some biological characteristics can be predictive markers of further PTSD development. For example, on the molecular level it has been shown that polymorphism in the FKBP5 (glucocorticoid-regulating co-chaperone of stress proteins) gene is a predictive marker.

PTSD can cause harmful effects on cognition, memory, and response to stimuli affecting specific regions of the brain. Observable changes in the prefrontal cortex, hippocampus, and amygdala were found under psychological trauma (Bremner, 2006). The amygdala is a core component of brain structure that is implicated in the pathophysiology of PTSD. Under stressful conditions the amygdala releases norepinephrine, that increases heart rate and, in this way, plays a key role in the regulation of emotions (Ousdal et al., 2020). Moreover, the prefrontal cortex (PFC) acts together with the amygdala to regulate emotions and behavior (Blair, 2008). High levels of cortisol that are released by the hypothalamus during stress are thought to damage hippocampal cells (Schmidt et al., 2020). PTSD patients with chronic stress typically show hippocampal damage, reducing the size and function if this brain region (Tull et al., 2020).

**FIGURE 1:**
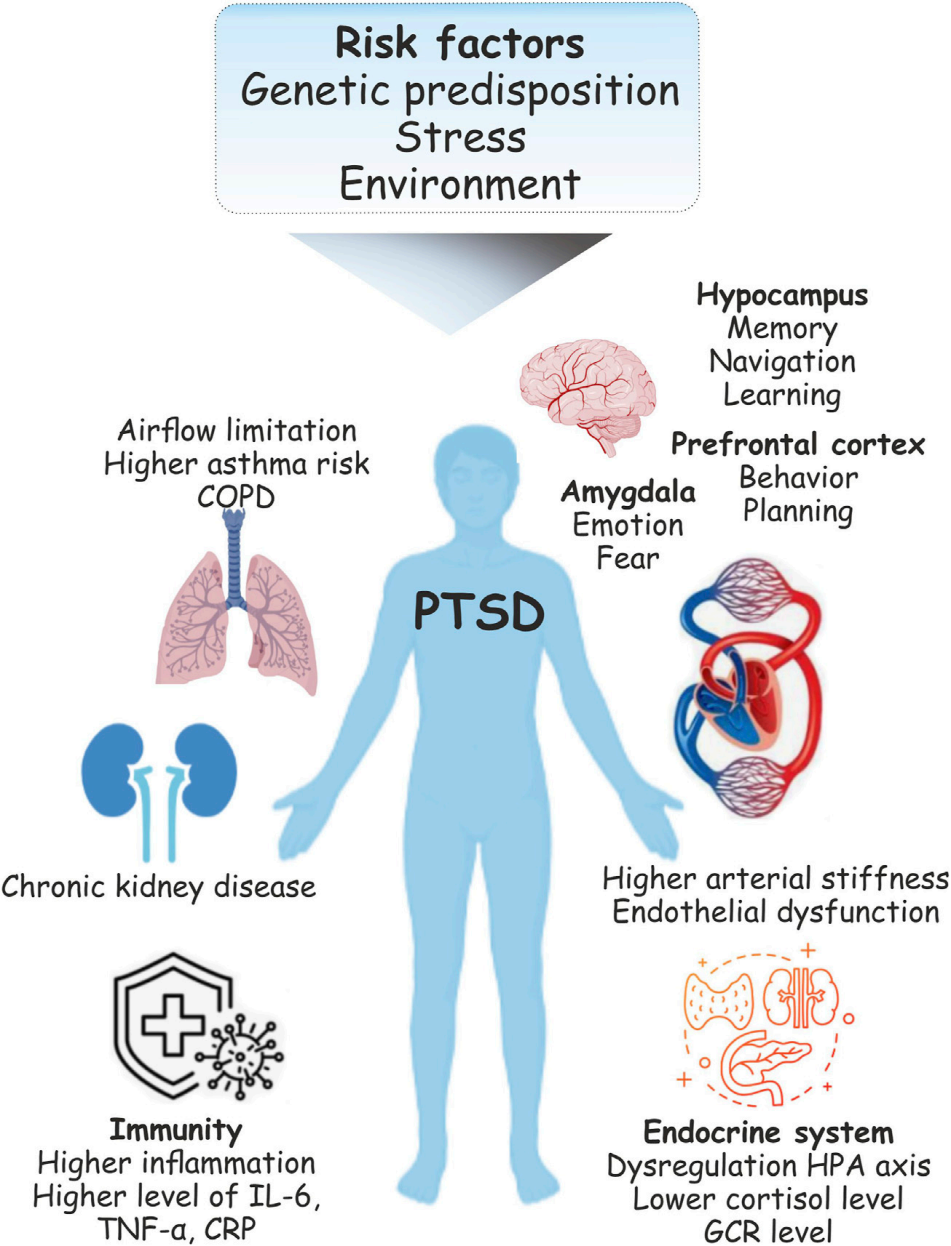
Physiological changes during post-traumatic stress disorder (PTSD). Following the traumatic event, PTSD is common and is one of the serious health concerns. PTSD can be associated with lasting changes in the brain areas implicated in the stress response include the amygdala, hippocampus, and prefrontal cortex. Functional limitations and respiratory syndromes may co-ocuure with PTSD. Stress is also the major risk factor for cardiovascular disease (CVD). Pathophysiology includes changes in the immune system function in individuals with PTSD suggest its association with enhanced immune inflammatory activity.

The pathophysiology of PTSD is also associated with major forms of cardiovascular disease including factors attributed to atherosclerosis such as coronary heart disease and thromboembolic stroke (Coughlin, 2011). Indeed, the brain-heart axis relationship has been implicated in a heightened risk of cardiovascular disease (CVD) during PTSD (Seligowski and Ressler, 2022). Patients with PTSD show over twice the risk of higher arterial stiffness and endothelial dysfunction than do unaffected people. Inflammation is one of the main biological mechanisms of increased CVD risk under PTSD (Brudey et al., 2015). Increased inflammatory biomarkers were found in patients with PTSD (Gill et al., 2009) including higher levels of IL-6, TNFα, and C-reactive protein (CRP) (Gill et al., 2009; Spitzer et al., 2010). Moreover, inflammatory processes also affect pulmonary diseases (Spitzer et al., 2011a). Several studies have also reported an impact of PTSD on obstructive pulmonary disease (COPD)-related outcomes (Abrams et al., 2015). Traumatic experiences can also cause respiratory conditions characterized by airflow limitation and asthma-related symptoms (Spitzer et al., 2011b). Stress related disorders (SRDs) including PTSD may also increase the risk of chronic kidney disease (CKD) progression and acute kidney injury (AKI) (Su et al., 2021) (Figure 1).

One of the greatest problem in the diagnosis and investigation of PTSD is the high level of comorbidities (Kessler, 2000). This can be attributed to a commonality of genetic origin (Koenen et al., 2009). For example, polymorphism of the serotonin transporter gene (STG) is present not only in PTSD, but in cases of major depression (Lee et al., 2005). Other widespread psychiatric syndromes—panic disorder and generalized anxiety disorder—are 60% identical in genetic origin (Chantarujikapong et al., 2001). Because the genes causing PTSD are also common to other widespread mental disorders, such as depression and anxiety, these other disorders can also be risk factors for PTSD development.

## 3 PTSD and neuroendocrine changes

There are three main groups of PTSD-induced changes in the neuroendocrinal system: functional alterations, structural aberrations and neurochemical changes. All of these abnormalities originate from malfunction of the hypothalamic–pituitary–adrenal (HPA) axis, prefrontal cortex, hippocampus, and amygdala, leading brain regions responsible for corresponding signal processing (Bremner et al., 2003). For example, the amygdala lateral nucleus is known to play a central role in triggering fear (Davis et al., 2003; Morey et al., 2012).

Abnormalities in the levels and activity of cortisol and thyroid hormones are key endocrine events in PTSD formation (Dekel et al., 2017). One of the central players in the response to stress is glucocorticoid hormone (GC). Concentrations of GC in blood plasma can reach their highest levels 20–40 min after activation of a stress factor, returning to normal after about 60 min (de Kloet et al., 2005). Effects of GCs on an organism’s physiology are very informative as a PTSD diagnostic. For example, dysregulation of the HPA axis can be confirmed by hypocortisolemia (low levels of cortisol) in blood plasma against a background of increased corticotropin-releasing hormone (CRH) in cerebrospinal fluid (Baker, 2005). HPA-dependent release of GCs under stress conditions results in inhibition of lymphocyte proliferation. As a result, levels of pro-inflammatory cytokines IL-6, IL-12, interferon γ (IFN-γ), and tumor necrosis factor α (TNF-α) are significantly decreased (Kim et al., 2020).

Dysregulation of estrogenic hormones may lead to methylation of histone deacetylase 4 (HDAC4) that is one of the risk factors in PTSD development. HDAC4 plays global roles in the regulation of gene transcription, cell growth, survival, and proliferation, and aberrant expression of HDAC4 activity leads to cancer development (Wang et al., 2020). Sensitivity to fear and its intensity depends on the state of HDAC4 methylation. Moreover, the hdac4 gene is methylated as more intensive PTSD develops and, *vice versa*, low methylation levels correlate with lower fear sensitivity (Maddox et al., 2018).

Dangerous conditions cause the release of adrenocorticotropic hormone (ACTH) by the pituitary that signals adrenal glands to secrete cortisol. Cortisol plays an important role in the modulation and regulation of metabolism (Baudrand and Vaidya, 2015). Furthermore, it accelerates PTSD development by renewal of traumatic memories and their recombination (Speer et al., 2019). Cortisol-conditioned cellular reactions mediate translocation of glucocorticoid receptors directly to the nucleus and regulate gene transcription in this manner (Ramamoorthy and Cidlowski, 2016). Corticotropin-releasing factor (corticoliberin, or CRH), a hypothalamic peptide stimulator of adrenocorticotropic hormone (ACTH) synthesis by the pituitary gland, is of great importance in PTSD pathogenesis with a direct correlation between the severity of PTSD, psychotic disorders, destructive personality disorders, and even suicide (Baker et al., 1999).

**FIGURE 2:**
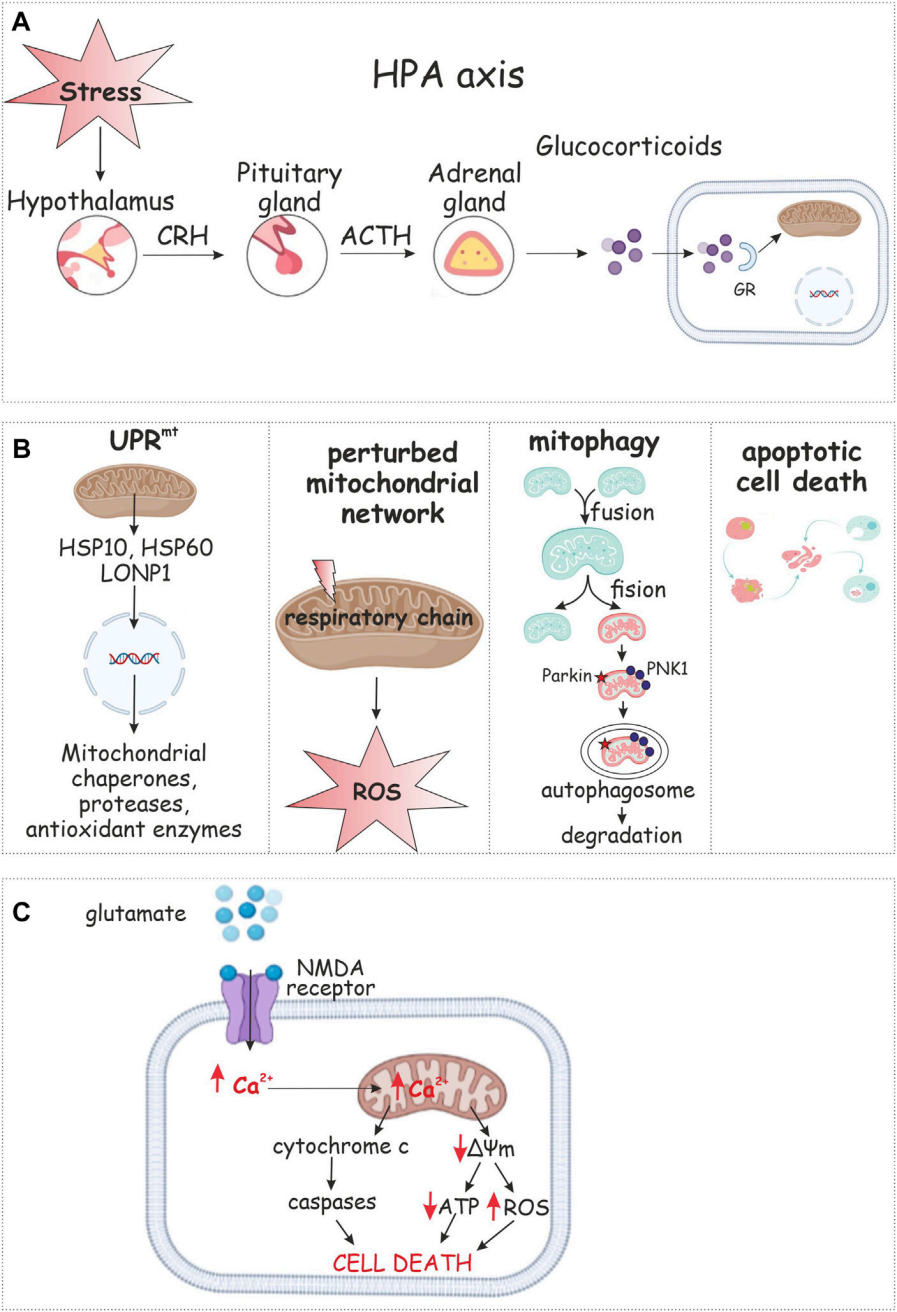
Mitochondrial response to PTSD. The hypothalamic–pituitary–adrenal (HPA) axis is the primary neuroendocrine pathway involved in stress response *via* release of secretions **(A)**. Glucocorticoids appear to regulate mitochondrial transcription. Mitochondrial stress response includes changes in mitochondrial dynamics, retrograde signaling, the mitochondrial unfolded protein response (UPR_mt_), selective autophagy of mitochondria (mitophagy), or apoptotic cell death **(B)**. Stress results in synaptic release of glutamate and binding to NMDA receptor that leads to calcium influx. Ca^2+^ overload results in collapse of mitochondrial membrane potential (ΔΨm), decrease in ATP production, and enhanced ROS generation **(C)**.

The HPA axis is the main coordinating element of the neuroendocrine response by mammals to stress (Pace and Heim, 2011). Some researchers have identified hypoactivity of the HPA against a background of sympathetic nervous system (SNS) hyperactivation. It is possible to hypothesize that these two response systems to stress act on PTSD development independently. The most widespread and regular responses of the neurohumoral system to PTSD that are currently known are a low response of the HPA and a hyper response of the catecholamine system. Avoidance behavior may be caused by a lower concentration of cortisol, whereas a higher catecholamine level is associated with reexperiencing stress and symptoms of hyperarousal (Geracioti et al., 2001). Extra-hypothalamic regions that influence the HPA axis through neuropeptide secretion include the bed nucleus of the stria terminalis (BnST), dorsal raphe nucleus (raphe), nucleus of the solitary tract (NTS), and the ventral subiculum (Vsub). The neurotransmitters from these regions can have inhibitory [γ-aminobutyric acid (GABA)] or excitatory [norepinephrine (NE), and serotonin (5-HT)] effects on the PVN (Arnett et al., 2016). Constantly high and stable levels of norepinephrine in blood accompanied by comparatively lower concentrations of GS confirm the hypothetical mechanism of response by the neurohumoral system to PTSD. Hence, stressful conditions induce secretion of corticotropin-releasing hormone (CRH) by neuron terminals of the hypothalamic paraventricular nucleus (PVN) to the hypothalamo-hypophyscal portal. CRH modulates and activates secretion of adrenocorticotropin (ACTH) from the anterior pituitary. ACTH provides GСc release from the adrenal cortex (Danan et al., 2021) (Figure 2A). GSc rebuilds metabolism to stress-responsive pathways, modulates functions of the brain and immune system and is sometimes called the «stress response orchestrating director» (Szeszko et al., 2018). A more acute course of PTSD with pronounced symptoms are associated with long-lasting high cortisol concentrations accompanied by lower reactivity to stress. It is possible that both hypersecretion of cortisol by the SNS and low secretion under the action of traumatic factors focuses the system towards PTSD development (Yehuda et al., 2002).

Allopregnanolone (ALLO) biosynthesis, as a pathophysiological mechanism that is involved in stress response under PTSD, has been highlighted in numerous reports (Agis-Balboa et al., 2014; Locci and Pinna, 2017; Dichtel et al., 2018). Downregulation of neurosteroid ALLO biosynthesis was observed in PTSD patients (Pibiri et al., 2008; Pinna and Rasmusson, 2012) and in mice exposed to prolonged (3–4 weeks) social isolation stress (Pinna, 2019). Regulation of stress response by ALLO realizes *via* allostatic mechanisms exerting negative feedback on the HPA axis (Almeida et al., 2021).

Some other humoral markers of PTSD development are also known. For example, levels of thyroid stimulating hormone (TSH) and prolactin are reduced in patients with PTSD (Olff et al., 2006). Some neuropeptides that are known to be responsible for social forms of behavior and neuroendocrine reactions to stress, like oxytocin (OT) and vasopressin (VP), are also associated with PTSD. However, the action of the OT-VP system under PTSD has been disrupted (Frijling et al., 2015).

## 4 Mitochondria and oxidative stress in PTSD pathophysiology

Metabolic and signaling processes within cells are accompanied by generation of reactive oxygen species (ROS) (Lushchak et al., 2021). All living organisms possess systems to protect against free radicals and these include low molecular mass antioxidants and proteins with enzymatic activities (Lushchak, 2015). An imbalance between the generation and elimination of ROS that favors generation is called “oxidative stress” and is associated with free radical attack on diverse cellular components (Lushchak et al., 2021). The most powerful source of oxidants in cells comes from the energy metabolism of mitochondria (Venditti et al., 2013). Mitochondria are double-membrane endosymbiotic cell organoids containing their own DNA (mDNA). Mitochondrial DNA encodes various proteins of the respiratory chain complexes I, III, IV, and V. Complex II, also known as succinate dehydrogenase (SDH) or succinate coenzyme Q reductase (SQR) is the only complex that is fully encoded by nuclear DNA (Lushchak, 2014). The ATP generated during oxidative phosphorylation inside mitochondria is exported *via* the inner mitochondrial membrane into the cytoplasm by adenine nucleotide translocators 1 and 2 (Cooper, 2000). Cytotoxic products resulting from the passage of electrons along the electron transport chain of the mitochondria are ROS. These have genotoxic, cytotoxic, and oxidative properties, and if antioxidant defense systems of cells are disrupted, ROS can lead to the development of oxidative stress (Lambert and Brand, 2009).

Oxidative stress and mitochondria have been implicated in several psychopathologies including PTSD. The human brain consumes approximately 25% of inhaled oxygen and is notably more susceptible to oxidative stress as compared with all other organs. This occurs because the brain contains significant amounts of polyunsaturated fatty acids (e.g., arachidonic, docosahexaenoic, etc.) (Bazinet and Layé, 2014) and a high level of redox transition metals (mainly Ca^2+^) (Garza-Lombó et al., 2018). Brain also has a lower levels of antioxidant defenses than do other organs; e.g., brain has 50-fold lower catalase activity in its neurons as compared to hepatocytes (Lee et al., 2020). The brain also needs extremely high levels of ATP for normal functioning and this requires a greater intensity of oxidative phosphorylation as compared with other organs and leads to an accompanying higher production of ROS (Lushchak et al., 2021). Oxidative damage to brain tissue can also be caused by hydrogen peroxide that is formed during endogenous neurotransmitter metabolism (dopamine, etc.) (Halliwell, 2006). These events eloquently emphasize an outstanding role of stress, ROS and mitochondria in the malfunctioning of brain that could contribute to PTSD as the result.

## 5 Mitochondria as PTSD coordinator and modulator

Mitochondria are the “first responders” of cell reactions to changing environmental conditions. The diversity of mitochondrial functions is exquisitely balanced, but this balance is easy to break and can rapidly lead to apoptosis, necrosis, ROS explosion, and various cellular abnormalities (Marchi et al., 2012) (Figure 2B). Higher membrane permeability, collapse of the mitochondrial membrane potential, release of complex IV of the ETC, and dysregulation or malfunction of the ETC are the main events in mitochondria under stress (Picard et al., 2018). These conditions significantly disrupt the normal course of oxidative phosphorylation, leading to abnormal (usually, significantly lower) ATP synthesis (Oroian et al., 2021).

N-methyl-D-aspartate (NMDA) mediates excitotoxicity and neuronal cell death or severe damage as can occur under stress and PTSD. This mechanism of neurotoxicity is mediated by mitochondria (Figure 2C). The NMDA receptor can show calcium-buffering activity, acting as an ion channel. NMDA-mediated neuronal activation depends on levels of Ca^2+^ buffered by mitochondria (Carvajal et al., 2016). This way of neuronal activation also plays an important role in mitochondrial-mediated oxidative stress initiation and progress *via* stimulating generation of ROS and reactive nitrogen species (RNS). These highly cytotoxic molecules are attributed to be responsible for neuronal cell death (Reynolds and Hastings, 1995). Mitochondrial stable ROS levels also mediate GABA-ergic inhibitory signaling (Accardi et al., 2014).

The stress response involves important changes in mitochondrial function. Stress responsive pathways enable mitochondria to sense environmental changes and regulate bioenergetic function providing ATP by oxidative phosphorylation, buffering Ca^2+^ and regulate apoptotic functions to maintain cellular homeostasis. Depending on stress intensity and duration, the mitochondrial stress response includes changes in mitochondrial dynamics, retrograde signaling, the mitochondrial unfolded protein response (UPR^mt^), selective autophagy of mitochondria (mitophagy), or apoptotic cell death (Whelan and Zuckerbraun, 2013) (Figure 2).

Mitochondria are principal regulators of cellular homeostasis under stressful conditions through integrated signaling of the UPR^mt^ (Fu and Zhang, 2017). The disruption of mitochondrial proteostasis, impairment of genes involved in diverse aspects of mitochondrial function, and reduction of mitochondrial import efficiency have been shown to activate the UPR^mt^ (Melber and Haynes, 2018). It was shown that stress-induced mitochondrial dysfunction that causes chromatin remodeling promote UPR^mt^ activation (Tian et al., 2016). Increased production of mitochondrial reactive oxygen species (mtROS) under stress leads to UPR^mt^ activation that induces nuclear gene expression of mitochondrial chaperones, proteases, and antioxidant enzymes to repair defective mitochondria (Hill and Van Remmen, 2014). The transcription factor ATFS-1 stimulate the expression of specific set of genes (Nargund et al., 2015). Newly synthesized proteins enter dysfunctional mitochondria within the cell to repair damaged organelles and restore functional activity. These proteins include chaperones [e.g., heat shock 10 kDa protein 1 (Hsp10), heat shock 60 kDa protein 1 (Hsp60)] (Martinus et al., 1996), which mediate the refolding of proteins into their proper conformation, and proteases [e.g., lon protease homolog (LonP1)], which degrade mitochondrial matrix proteins, which accumulate as electron-dense inclusions (Suzuki et al., 1994). The UPR^mt^ genes may be used as biomarkers for the mitochondrial disease under exposure to many of the stressors (Suomalainen et al., 2011).

Mitochondrial dysfunction also leads to a great release of mtROS that can trigger apoptosis (Guo et al., 2013). Prolonged stress in animal models of PTSD was shown to lead to atypical apoptosis in the hippocampus, the amygdala, and the medial prefrontal cortex (Jia et al., 2018). Mitochondrial morphological changes or dysfunction appear to be critical to cell health. Elevated mtROS level results in oxidative damage of proteins, lipids, and nucleic acids that may contribute to the progression of PTSD. Damaged mitochondria can be degraded *via* a process known as mitophagy and this can be triggered by moderate levels of mtROS. Mitophagy significantly decreases ROS levels, ATP production, and prevents mtDNA accumulation (Bakula and Scheibye-Knudsen, 2020). PTEN-induced kinase 1 (PINK1), parkin, and protein deglycase DJ-1 are the main proteins that trigger mitophagy (Fivenson et al., 2017). A decline in mitophagy leads to mitochondrial dysfunction.

Mitochondria can coordinate and modulate key physiological and biochemical processes that can ultimately lead to the development of PTSD. Dysfunction or malfunction of so-called «suboptimal mitochondrial function» (SMF) are known to be closely related to mental disorders and psychopathologies including PTSD. Chronic and acute stress exposures alter specific aspects of mitochondrial structure and function (Picard and McEwen, 2018). Mitochondrial swelling and membranes distention occur under stressful conditions (Picard and McEwen, 2018). The swelling of mitochondria is regulated by Ca^2+^ and K^+^ influx/efflux. Mitochondrial matrix swelling results in the opening of mitochondrial permeability transition pores (PTP), and disruption of mitochondrial membrane integrity (Javadov et al., 2018). Malfunction or disfunction of mitochondria has been linked to conditions including depression, schizophrenia, chronic disturbances and PTSD (Pei and Wallace, 2018). Connections between mitochondria and mental health may be implemented by changes in monoamine oxidase A and B enzyme activities. The Peripheral Benzodiazepine Receptor (PBR) of mitochondria was shown to be involved in the processes relevant to PTSD pathogenesis including mitochondrial transmembrane potential, its sensitivity to ROS, and neurosteroid synthesis (Casellas et al., 2002; Pei and Wallace, 2018).

It was identified that the mitochondria-focused genes underlying the pathogenesis in PTSD-related brain regions using a recently developed third generation human mitochondria-focused cDNA microarray (hMitChip3) (Su et al., 2008). The study of Su et al. (2008) found PTSD-specific expression fingerprints of 800 informative mitochondria-focused genes in postmortem brain of patients with PTSD. Moreover, 119 dysregulated genes were associated with mitochondrial dysfunction including oxidative phosphorylation, cell survival-apoptosis and neurological diseases (Su et al., 2008). Examination of profiles of mitochondria-focused gene expression in stressed-rodent model (inescapable tail shock in rats), which shows characteristics of PTSD-like behaviors demonstrated that 34 mitochondria-focused genes are upregulated in stressed-rat amygdala (Zhang et al., 2015). Carnitine palmitoyltransferase 1B (CPT1B), an enzyme in the fatty acid metabolism and peroxisome proliferator-activated receptors (PPAR) pathways was significantly over-expressed, not only in the amygdala and in the blood of stressed rats but also in the blood of PTSD patients (Zhang et al., 2015). Dysregulated mitochondria-focused genes present in both rodent stress model and postmortem brains of PTSD patients might serve as PTSD-related biomarkers (Su et al., 2008).

## 6 Mitochondria, inflammation and PTSD

Investigations of PTSD have proven that changes in the immune system are prominent and key symptoms of this syndrome. These alterations are usually manifested by an activation and elevated concentrations of pro-inflammatory cytokines (IL-1β, TNFα, IL-2, IL-6, IL-17), interferon gamma (INF-γ), and C-reactive protein (CRP) as well as a decrease in IL-4 levels, disrupting normal immune cell balance (Lopez-Armada et al., 2013).

Inflammatory processes are closely connected to mitochondria. ROS production is the first signaling step in the inflammatory response. Mitochondrial abnormalities, resulting in ROS overproduction, activate the secretion of IL-1β (Strowig et al., 2012). If levels of ROS are too high, mitoptosis can occur and mitochondrial DNA (mtDNA) can be broken into smaller fragments. These degraded elements of mtDNA can induce an increase in IL-1β, IL-6, and TNF concentrations in mice (Mathew et al., 2012). Release of mtDNA is possible due to inflammasome activity and inhibited by NALP3 (cryopyrin) (Nakahira et al., 2011).

Some pro-inflammatory cytokines are able to influence oxidative phosphorylation. For example, IL-1β and TNFα can downregulate and inhibit complex I activity and synthesis of ATP. This leads to activation and notable increases in ROS production (Guidarelli et al., 2007). Activity of amygdala neurons increases by enhanced activity of stress-mediated IL-6 (Ventrano, 2017). This interleukin is known to disturb mental health and enhance the activity of the subgenual (“subcallosal”) anterior cingulate cortex (subgenual ACC) with underlying neuroinflammation (Eslinger et al., 2021). IL-6 also shows an ability to increase dopamine (DA) in the amygdala and hippocampus and, in this way, modulate fear learning and processing of stress situations (Pervanidou et al., 2007). Furthermore, IL-6 levels are able to dysregulate the HPA axis normal activity, stimulating in this way the development of PTSD symptoms.

## 7 Conclusion

PTSD is a widespread trauma-associated polyetiological long-lasting or chronic complex of molecular, cellular, and systemic disorders. In general, PTSD leads to disruption of energetic and plastic metabolism, and pathologies of nervous, cardiovascular, endocrine, and immune systems, that manifest into mental diseases, transformation of personality, significant deterioration in health, and a noticeable decrease in the quality of life. One of the first stress reactions in the resulting physiological responses are neurohumoral responses from the HPA axis. Acute psychological stress modulates levels and metabolism of glucose, lipids, and amino acids. These biochemical changes possibly cause the chronic form of PTSD. Internal metabolic and genetic factors determine rate, character and scale of the final cellular and organism reactions, resulting in psychological changes or psychiatric syndromes. To a major extent, mitochondria modulate the effects of psychological stress on metabolic perturbations. Mitochondria act as a director, processor and interpreter of general and cellular stress signaling and are able to modulate subsequent systemic reactions. Furthermore, mitochondria may violate normal interactions among stress-reactive organs, their functional systems and cells themselves. Mitochondrial malfunctions and disorders play determining roles in vulnerability to PTSD, its severity, development and progression to a chronic form. Closer, more intensive and diversified cooperation between scientists, PTSD treatment researchers and physicians is needed to summarize all current experimental data and determine the role of individual molecules and their complexes, organs and their systems, internal and external factors that may enhance or weaken the development of PTSD. This can lead to developing a single, adequate, multifaceted and effective strategy for correcting or overcoming PTSD. Novel therapeutic agents should be developed to reduce neuro-inflammation and improve mitochondrial physiology in neurons to benefit the PTSD patients.
